# Thickness of Actinic Keratosis Does Not Predict Dysplasia Severity or P53 Expression

**DOI:** 10.1038/srep33952

**Published:** 2016-09-27

**Authors:** Ida M. Heerfordt, Christoffer V. Nissen, Thomas Poulsen, Peter A. Philipsen, Hans Christian Wulf

**Affiliations:** 1 Department of Dermatology, Bispebjerg Hospital, University of Copenhagen, DK-2400 NV Copenhagen, Denmark; 2 Department of Pathology, Hospital of Southern Jutland, DK-6400 Soenderborg, Denmark

## Abstract

The severity of dysplasia and expression of p53 in actinic keratosis (AK) is of importance for the transformation to squamous cell carcinoma. It is assumed that it is most important to treat thick AKs as they are believed to be more dysplastic than thin AKs. However, a relation between AK thickness and dysplasia or the expression of p53 has never been demonstrated. The aim of this study was to investigate this possible relation. Sixty-six AKs were included for clinical and histological examination. Prior to performing a punch biopsy, the clinical thickness of each AK was measured objectively using two scale bars with a thickness of 0.5 mm and 1 mm. Subsequently, the thickness of the epidermis, the severity of dysplasia and the expression of p53 were assessed histologically. We found a strong and significant positive correlation between measured clinical thickness of the AKs and the histological thickness of epidermis (p < 0.0001). However, the clinical thickness did not correlate with either the severity of dysplasia (p = 0.7) or the expression of p53 (p = 0.5). In conclusion, thin AKs show the same severity of dysplasia and expression of p53 as thicker AK lesions. Consequently, clinical thickness cannot predict aggressiveness.

Actinic keratosis (AK) is a precursor of squamous cell carcinoma (SCC)^1^,^2^. Studies show that p53, the most frequently mutated gene in human cancers, is mutated in up to half of AKs and more than half of SCC^3^,^4^,^5^. Mutations in the p53 gene can lead to increased expression of p53 protein in the tissue and ultimately cancer^6^,^7^. The severity of dysplasia assessed histologically as classified by Rowert-Huber *et al*.^8^ is also of importance for the transformation of AKs into SCCs as it occurs through a progression from mild to moderate to severe dysplasia and ultimately to SCC^1^,^8^,^9^,^10^,^11^,^12^,^13^,^14^,^15^,^16^,^17^. The expression of p53 in the various severities of dysplasia has not been investigated so far.

It is believed that severe dysplastic AKs are thicker than mild dysplastic AKs^1^,^9^,^10^,^11^,^12^,^13^,^14^. Consequently, it is a priority to remove thick AKs^13^,^18^. However, this relation between clinically assessed thickness and dysplasia has never been demonstrated.

Assessing AK thickness is controversial. Olsen *et al*.^19^ proposed a classification system based on a subjective assessment of the overall thickness of AKs on each patient. This classification system has since been widely used in a modified version^20^. However, the completely subjective nature of the classification system makes it difficult to compare results from different studies, and currently there are no approved objective methods for clinical classification of AKs^13^.

We used two alternative objective methods to measure AK thickness; (i) measurement of clinical thickness with custom made scale bars which were placed on the skin next to the AK to determine how much the keratosis protruded above normal skin; and (ii) measurement of the stratum corneum hydration of each AK. The stratum corneum hydration was used as a surrogate measurement of AK thickness because hydration gradually decreases in keratotic tissue.

The aim of this study was to establish an objective clinical classification system of AKs which corresponds well with the histological thickness. Moreover, we aimed to investigate whether AK thickness correlates with dysplasia or expression of p53.

## Results

Seventy lesions were clinically and histologically investigated. Histology confirmed 66 lesions as AKs, while one was a squamous cell papilloma and three were hyperkeratoses without histological dysplasia. Lesions not histologically diagnosed as AKs were excluded. The AKs were sampled from the trunk (n = 21), upper limbs (n = 37) and lower limbs (n = 8).

### Clinical thickness

**Figure 1:**
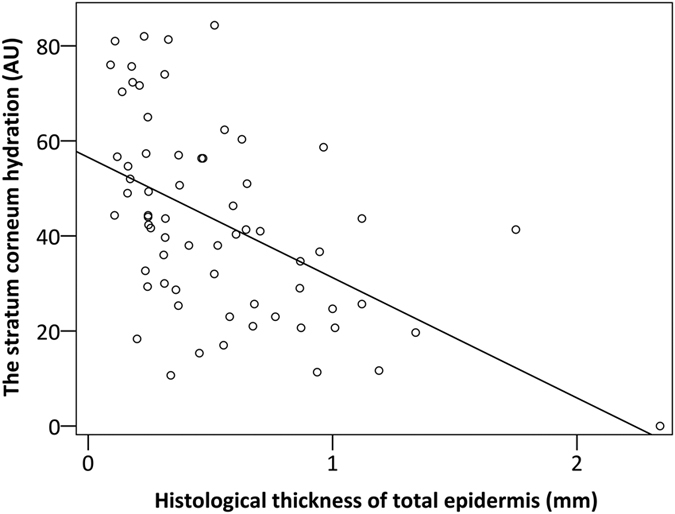
The stratum corneum hydration and corresponding histological thicknesses. Scatter plot of the stratum corneum hydration and corresponding histological thicknesses of total epidermis. The stratum corneum hydration decreased significantly with increasing histological thickness of total epidermis (r = 0.54, p < 0.0001).

The clinical thickness was differentiated in three categories; (i) less than 0.5 mm;(ii) between 0.5 and 1 mm; and (iii) more than 1 mm. The histological thickness of the stratum corneum, cellular epidermis and total epidermis of the 66 AKs are shown in Table 1. The histological thickness of the stratum corneum was significantly different in the three categories of clinical thickness (in all comparisons, p < 0.0002), and stratum corneum thickness increased significantly with increasing clinical thickness of AK (r = 0.68, p < 0.0001) as did the histological thickness of the cellular epidermis (r = 0.41, p = 0.001) and total epidermis (r = 0.72, p < 0.0001). The histological thickness of the stratum corneum increased significantly with increasing thickness of the cellular epidermis (r = 0.43, p = 0.003). The stratum corneum hydration decreased significantly with increasing histological thickness of the stratum corneum (r = 0.50, p < 0.0001), cellular epidermis (r = 0.33, p = 0.007) and total epidermis (r = 0.54, p < 0.0001) (Fig. 1). The clinical thickness was not patient dependent (p = 1.0).

### Dysplasia

**Figure 2:**
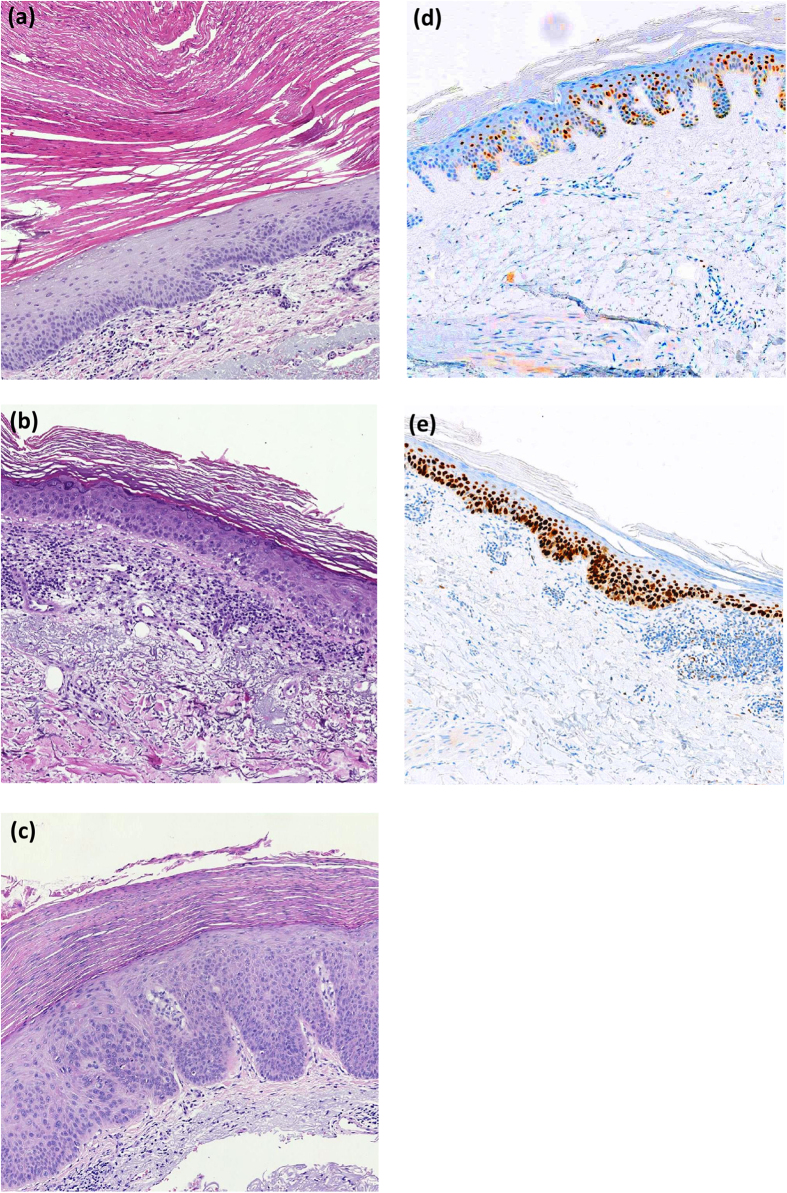
Histopathological examples. (**a–c**) Hematoxylin-eosin stain. (**a**) Mild dysplasia. (**b**) Moderate dysplasia. (**c**) Severe dysplasia. (**d,e**) P53 immunostain. Positive nuclei are brown, whereas negative nuclei are blue. (**d**) Low expression of p53. (**e**) High expression of p53.

**Figure 3:**
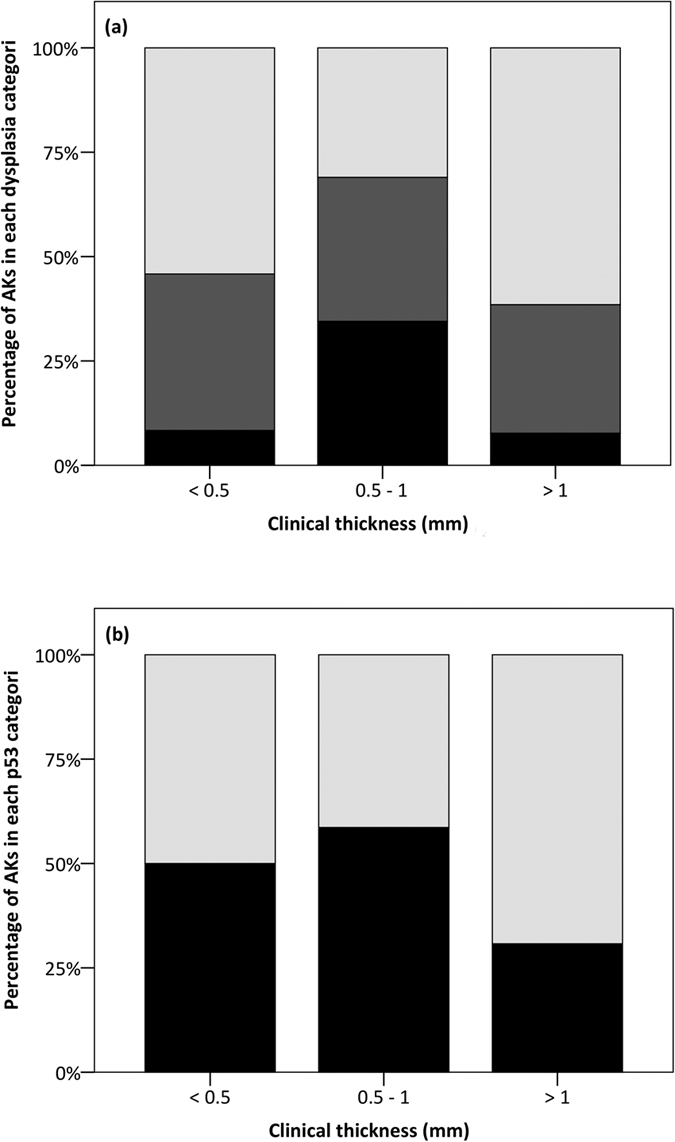
Severity of dysplasia, expression of p53 and clinical thickness. (**a**) Stacked bar chart of the severity of dysplasia (mild dysplasia coloured light grey, moderate dysplasia coloured dark grey and severe dysplasia coloured black) for the different categories of clinical thickness. We found no correlation between the severity of dysplasia and clinical thickness (p = 0.7). (**b**) Stacked bar chart of the expression of p53 (low expression of p53 coloured light grey and high expression of p53 coloured black) in the different categories of clinical thickness. We found no correlation between the expression of p53 and clinical thickness (p = 0.5).

Thirty AKs showed mild dysplasia, 23 showed moderate dysplasia and 13 showed severe dysplasia. Histopathological examples of mild, moderate and severe dysplasia are presented in Fig. 2. The severity of dysplasia in AKs with different clinical thickness is shown in Fig. 3a. We found no correlation between dysplasia severity and clinical thickness (p = 0.7).

Likewise, we found no significant correlation between the severity of dysplasia and the histological thickness of the stratum corneum (p = 0.10). Nor was there any significant correlation between the severity of dysplasia and the histological thickness of cellular epidermis (p = 0.11) or total epidermis (p = 0.10).

There was no relation between severity of dysplasia and gender (p = 0.9), age (p = 0.2), anatomical localization (p = 0.6), the stratum corneum hydration (p = 0.7) or the largest diameter of the AK (p = 0.10). The severity of dysplasia was not patient dependent (p = 0.6).

### Expression of p53

The expression of p53, classified as either low or high, in AKs with different clinical thickness is shown in Fig. 3b. We found no correlation between expression of p53 and clinical thickness (p = 0.5), histological thickness of the stratum corneum (p = 0.3), cellular epidermis (p = 0.6) or total epidermis (p = 0.6). The trend was that the expression of p53 increased with increasing severity of dysplasia. Only 43% of AKs showing mild dysplasia had a high expression of p53, whereas 48% of AKs with moderate dysplasia and 69% of AKs with severe dysplasia had a high expression of p53. The trend was, however, not statistically significant (p = 0.18).

There was no relation between expression of p53 and gender (p = 0.6), age (p = 0.2), anatomical localization (p = 0.8), the stratum corneum hydration (p = 0.8) or the largest diameter of the AK (p = 0.8).

## Discussion

The main reason for treating AKs is to prevent malignant transformation. AKs can be treated with different treatment modalities and much research has been done to prove efficacy^21^. AK thickness has an impact on treatment success rate, since thin lesions are easier to cure than thick ones^22^. Consequently, it is important that thickness is accurately measured in order to compare treatment efficacy in different studies^13^. Currently there is no objective method for clinical classification based on thickness.

We used two alternative objective methods to measure AK thickness: (i) measurement with scale bars and (ii) measurement of stratum corneum hydration. Both methods were significantly correlated with the histological epidermis thickness. Measurement with scale bars was however most strongly correlated with histological thickness and the simplest method to use. We therefore propose this as a new objective classification system.

Interestingly, neither clinical thickness nor histological thickness correlated to the severity of dysplasia. This was surprising since such a correlation has been widely assumed^1^,^9^,^10^,^11^,^12^,^13^,^14^. Our results are consistent with a recent study of Schmitz *et al*.^23^. They conclude that it is not possible to foresee the severity of dysplasia from the clinical appearance of the AK. The study used entirely subjective assessments of the clinical presentations and it is not known how thick the examined AKs were.

Transformation of AKs into SCCs is assumed to occur through a progression from mild to moderate to severe dysplasia and ultimately SCC^1^,^8^,^9^,^10^,^11^,^12^,^13^,^14^,^15^,^16^,^17^. Consequently, lesions with mild dysplasia are assumed to have low malignancy risk, because they require evolution to more advanced stages before obtaining dermal infiltrating capacity. Conversely, AKs with severe dysplasia are thought to involve a higher risk, as they correspond to the final stage preceding invasion. These assumptions are based on a histopathological examination of more than 1,000 SCCs contiguous with AKs, which found AKs with severe dysplasia at the periphery of the SCCs in 97% of the tumours^12^. However, when dysplasia is present it always starts close to the basement membrane and Fernandez-Figueras *et al*.^24^ suggest that penetration may possibly be independent of the degree of dysplasia. Our results, along with the findings of Fernandez-Figueras put the biological significance of the degree of dysplasia into question.

Our study is the first to investigate the relation between expression of p53 and severity of dysplasia as well as the relation between expression of p53 and clinical thickness. The results of our study show a trend towards increased expression of p53 with increasing severity of dysplasia. On the other hand, there is no correlation between clinical thickness and expression of p53.

Our study suggests that all AK lesions independent of their clinical thickness are equally invasive. Very thin slightly scaling AKs cannot safely be ignored, as has previously been recommended^13^,^18^, and the assumption that thin AKs should be treated for cosmetic reasons only cannot be maintained. In conclusion, there is a strong correlation between the clinical and histological thickness of AKs. However, severity of AK dysplasia and expression of p53 is independent of clinical and histological thickness. Thus clinical thickness of AKs cannot predict its aggressiveness.

## Methods

### Patients

Eligible patients were 18 or older and were clinically diagnosed with AKs. Patients were ineligible if they were pregnant or breastfeeding. Twenty-four patients were included. The group included 15 men and 9 women with ages ranging from 53 to 89. Each patient had two or three AKs examined. All patients provided informed written consent.

### Study design

For each AK the anatomical localization was noted. Subsequently, we measured the largest diameter, the clinical thickness and the hydration of the stratum corneum of each AK. The diameter was measured with an electronic caliper (Electronic digital caliper, Staco, Alleroed, Denmark), while the clinical thickness was measured using two scale bars produced for this purpose with a thickness of 0.5 mm and 1 mm. The bars were placed on the skin next to the AK to determine how much the keratosis protruded above normal skin. This made it possible to divide clinical AK thickness into three categories: (i) less than 0.5 mm; (ii) between 0.5 and 1 mm; and (iii) more than 1 mm. In reproducibility tests the intra-observer agreement of the scale bars measurement was found to be substantial (kappa value = 0.8), whereas the inter-observer agreement was moderate (kappa value = 0.5)^25^. The hydration of the stratum corneum was measured by performing a non-invasive capacitance measurement (Corneometer 820, Courage + Khazaka, Cologne, Germany) as described by Heinrich *et al*.^26^. The calibration of the Corneometer was checked every month by measuring hydration on three blocks of oasis (OASIS^®^ Grande Brick, OASIS Floral Products, Kent, USA) completely soaked in bath oil (Klinion oil, Mediq Medeco, Oud-Beijerland, The Netherlands), sterile water and ethanol 96% v/v, respectively. Measurements on these three liquids with different water content were stable at three different levels.

Finally, a 3 mm punch biopsy was taken from the thickest part of each lesion in infiltrative anaesthesia (carbocain with adrenaline, 20 mg/mL + 5 microgram/mL, AstraZeneca, London, Great Britain). The biopsy specimens were formalin-fixed and paraffin-embedded. After staining with hematoxylin and eosin evaluation was performed by a certified histopathologist blinded to the clinical investigation. Firstly, the lesions were histologically diagnosed as AKs or not. Secondly, the severity of dysplasia was classified histologically as proposed by Rowert-Huber *et al*.^8^ in the following way: mild dysplasia: atypical keratinocytes in the basal and suprabasal layers of the epidermis; moderate dysplasia: atypia involving the lower two-thirds of the viable epidermis; severe dysplasia: atypical keratinocytes in more than two-thirds of the full thickness of the viable epidermis. Thirdly, the histological thickness of the stratum corneum, cellular epidermis and total epidermis was measured as the maximal vertical thickness where the epidermis was thickest. Finally, the sections were stained for p53 protein using Anti-p53 (Bp53-11) primary antibody (Ventana Medical Systems, Roche Diagnostics) and the expression of p53 was asseded through the whole cellular epidermis and quantified as the percentage of p53 positive nuclei. Since the median percentage of p53 positive nuclei was 54%, AKs where more than 54% of nuclei were p53 positive were considered to have a high expression of p53. The rest were considered to have a low expression of p53.

The study was approved by the Ethics Committee of Region Hovedstaden, Copenhagen, Denmark (H-15001096) and the methods were carried out in accordance with this approval at the Department of Dermatology, Bispebjerg Hospital from April to June 2015.

### Statistics

No previous studies have investigated a possible relation between clinical or histological thickness and severity of dysplasia or expression of p53. Hence, the sample size requirement was based on a study of AKs where the mean histological thickness was found to be 0.82 mm with a standard deviation of 0.47 mm^27^. To be able to detect a difference between thin AKs with an expected mean histological thickness of 0.41 mm and thick AKs with an expected mean histological thickness of 0.82 mm, we needed 21 AKs in each group to reach a significance level of 5% and a power of 80%. In addition we included the thickest AKs in a separate group. To accommodate possible loss of samples during analysis we included 70 biopsies. If the effect of the thickness of AKs on severity of dysplasia or expression of p53 is too small to be detected on analysis of 70 AKs, its clinical relevance is of low impact.

The statistical analysis was performed in IBM SPSS statistics version 22.0.0 (IBM, Armonk, NY, USA). Since data were not normally distributed, only nonparametric tests were used. The Mann-Whitney test was used to test unpaired data. For relations between categorical data, Fisher’s exact test was used, and for correlations Spearman’s rank-order correlation was used. Concordance was assessed using kappa statistics. P-values less than 0.05 were considered significant.

## Additional Information

**How to cite this article**: Heerfordt, I. M. *et al*. Thickness of Actinic Keratosis Does Not Predict Dysplasia Severity or P53 Expression. *Sci. Rep.* **6**, 33952; doi: 10.1038/srep33952 (2016).

## Figures and Tables

**Table 1:** Clinical and histological thickness.

**Microscopic measurements**	**Clinical thickness**					
	**<0.5 mm (n = 24)**		**0.5–1 mm (n = 29)**		**>1 mm (n = 13)**	
	**Mean (SD)**	**Range**	**Mean (SD)**	**Range**	**Mean (SD)**	**Range**
Thickness of the stratum corneum (mm)	0.16 (0.13)	0.02–0.5	0.35 (0.21)	0.1–0.9	0.88 (0.52)	0.2–2.0
Thickness of the cellular epidermis (mm)	0.12 (0.05)	0.1–0.3	0.14 (0.09)	0.1–0.5	0.24 (0.16)	0.1–0.6
Total epidermis thickness (mm)	0.27 (0.17)	0.1–0.7	0.49 (0.21)	0.2–1.0	1.12 (0.50)	0.3–2.3

The clinical thickness of AKs and the histological thickness of the stratum corneum, cellular and total epidermis. The histological thickness of total epidermis increased significantly with increasing clinical thickness of AK (r = 0.72, p < 0.0001). Abbreviations: SD = standard deviation.
